# Sialendoscopy in Management of Juvenile Recurrent Parotitis—A Single Centre Experience

**DOI:** 10.3390/children9111632

**Published:** 2022-10-27

**Authors:** Luka Pušnik, Anže Jerman, Jure Urbančič, Aleksandar Aničin

**Affiliations:** 1 Faculty of Medicine, University of Ljubljana, 1000 Ljubljana, Slovenia; 2 Department of Otorhinolaryngology and Cervicofacial Surgery, University Medical Center Ljubljana, 1000 Ljubljana, Slovenia

**Keywords:** minimally invasive surgery, sialendoscopy, juvenile recurrent parotitis, otorhinolaryngology, salivary gland disease

## Abstract

Juvenile recurrent parotitis is a rare inflammatory disease of the parotid gland that shares diverse therapeutic management between institutions. Sialendoscopy has been demonstrated as an efficient diagnostics and therapeutic method with minimal complications; however, due to the rarity of the disease and limited data, there is a lack of universal guidelines on its optimal management. Herein, we retrospectively analysed patients with juvenile recurrent parotitis who had the sialendoscopy performed at our tertiary centre. Descriptive data were retrieved along with the number of swelling episodes one year before and after the sialendoscopy intervention. In the last decade, twenty-nine sialendoscopic procedures were performed at our clinics on twenty-one patients diagnosed with juvenile recurrent parotitis. Most of them underwent the procedure under general anaesthesia (86%). In the year before and after the sialendoscopic procedure, the patients had 3.9 ± 2.7 and 0.2 ± 0.4 episodes of swelling per year, respectively. The difference proved to be statistically significant (*p* < 0.0001). The complete resolution was noted in sixteen patients (76%); however, the procedure was not repeated on the same side of any patient. Solely one patient had a relapse of the disease reported more than twelve months after the sialendoscopy, nonetheless, one of his exacerbation episodes was already reported in the first year after the sialendoscopy. The mean follow-up period of patients was 48.6 months (range, 13–116 months). All things considered, this study emphasises sialendoscopy as an effective minimally invasive diagnostic and therapeutic tool for the management of juvenile recurrent parotitis.

## 1. Introduction

Juvenile recurrent parotitis is an inflammatory disease, often associated with non-obstructive sialectasia, that affects the parotid gland. Although considered rare, it is the second most common inflammatory disease of salivary glands in children. It is characterised by painful and intermittent swelling of the salivary glands that can be uni- or bilateral [1,2]. The condition is accompanied by overlying skin erythema, malaise, and pyrexia. The patients are typically affected with an acute episode for 3–7 days and on average 4–5 times a year. The frequency of attacks ordinarily ceases after puberty; however, the disease rarely continues in adulthood [3,4]. As frequent recurrences significantly reduce the quality of life and lead to progressive gland destruction, early recognition of the disease remains crucial [4,5].

The diagnosis of juvenile recurrent parotitis is based on clinical examination, and at least two recurrent episodes of parotitis are required for the diagnosis. With being the diagnosis per exclusionem, the inflammation caused by viral and bacterial pathogens together with Sjögren syndrome and sialolithiasis ought to be carried out [4,6]. The clinical confirmation is generally supported with radiological imaging; nonetheless, this varies immensely across institutions [5,6,7]. Ultrasound (US) of the salivary glands is frequently used in the paediatrics population as it can insonate cystically enlarged ducts that represent as several small and round hypoechoic areas, enlarged intraparotid lymph nodes, or microcalcifications [8]. In acute phasis, hypervascularity can be detected with Doppler mode. The US is limited with spatial resolution, especially in the visualisation of smaller ducts and their sialoliths, and by being a solely diagnostic technique [5]. Contrarily, other radiological techniques such as sialography can be used as therapeutic methods. In the sialography, radiopaque contrast is injected into the duct and the salivary gland’s architecture can be depicted up to fourth-order branching [9]. The latest is, however, an invasive procedure limited by radiation exposure, possible allergic reactions, and distal duct obstructions. In past decades, sialendoscopy as a relatively new and minimally invasive technique for the diagnosis of salivary diseases has been proposed [10]. The endoscopic procedure enables observation of the anatomical course of salivary ducts, evaluation of other possible pathologies, and further management. Therefore, sialendoscopy portrays a new frontier as the therapeutic tool for the minimally invasive treatment of juvenile recurrent parotitis enabling dilatation of the main duct, washout of mucus plugs/cellular debris, and instillation of anti-inflammatory medications [11].

Hitherto, several researchers have shown a significant impact of sialendoscopy on alleviating the symptoms of juvenile recurrent parotitis [3,12,13,14]; however, due to the scarcity of this disease, more data are still required for establishing global consensus on its optimal management. Our study aimed to present a long-term tertiary centre experience with sialendoscopy in the management of juvenile recurrent parotitis. In detail, we retrospectively analysed patients who underwent the procedure and compared the number of juvenile recurrent parotitis episodes one year before and after the sialendoscopy. Moreover, we aimed to present a longer follow-up period after the sialendoscopy compared to formerly published literature.

## 2. Materials and Methods

### 2.1. Ethical Approval

The study was approved by National Medical Ethics Committee (reference number 0120-80/2017/4 and the approval date is 14 March 2017). The majority of patients in this study were children, therefore the informed consent was signed by their legal guardians. The participants, however, gave their informed assent to the study. With written consent, the parents agreed to using patients demographical/clinical data in future research. The research was conducted following the principles of the Declaration of Helsinki.

### 2.2. Patients and Data Retrieval

This study retrospectively reviewed patients diagnosed with juvenile recurrent parotitis who were treated endoscopically at the Department of Otorhinolaryngology and Cervicofacial Surgery of the University Medical Clinical Centre Ljubljana between September 2011 and September 2021. The inclusion criteria were: two or more acute episodes of parotid gland swelling on one or both sides, unresponsiveness to conventional non-steroidal anti-inflammatory drugs, onset of a disease before the age of sixteen, and performed sialendoscopy of at least one salivary gland. The exclusion criteria were: non-cooperation of the patient, presence of obstructive lesions (i.e., sialolithiasis), and Sjögren syndrome.

Descriptive data were obtained to specify patients’ characteristics. For each patient, we gathered information on the number of acute episodes of juvenile recurrent parotitis one year before and in the period after the sialendoscopic procedure. The data about the peri- and post-operative complications, length of hospitalisation, US appearance of the parotid gland, the endoscopic appearance of Stenson’s duct, and presence of stenosis were also collected. The latest was classified according to the LSD (lithiasis, stenosis, dilatation) classification [15]. In brief, S0 labels the complete absence of stenosis; S1 unique or multiple intraductal diaphragmatic stenosis; S2 unique ductal stenosis of the main duct; S3 multiple or diffuse ductal stenosis of the main duct; and S4 represents the narrowing of all ducts. The complete resolution of disease was defined as an absence of swelling of the treated parotid gland in the period one year after the procedure.

### 2.3. Sialendoscopic Procedure

The patients were examined by an otorhinolaryngology specialist, who confirmed the diagnosis of juvenile recurrent parotitis based on the clinical criteria and indicated the sialendoscopy. The endoscopic procedure was performed under general anaesthesia for the patients aged less than fifteen, and the patients who were older than fifteen and compliant underwent the intervention under local anaesthesia. 

Following the perioperative preparation, the sialendoscopy was done to define the ductal pathology followed by an intervention procedure in the same sitting with the instillation of steroids in solution. The first step involved dilating the papilla using salivary probes of increasing diameter (Marchal dilator system, Karl Storz, Tuttlingen, Germany). The dilatated papilla served for the introduction of the sialendoscope. The endoscope type Erlangen or type Marchal (Karl Storz, Tuttlingen, Germany) with an external diameter of 0.89 mm or 1.1 mm was introduced. Continuous lavage of saline solution with local anaesthetic (0.5% lidocaine solution) was performed during the intervention. The gentle irrigation of the solution maintained the duct open and favoured the removal of plugs and cell debris. In the event of a markedly narrowed ductus, the operator changed the endoscope to a smaller calibre or used the guiding wire to overpass the obstruction. After the lavage, dexamethasone (4 mg in 1 mL solution) was irrigated into the main duct. If both parotid glands were affected, the procedure was repeated on the contralateral side. After the procedure, each patient received oral prophylactic antibiotic therapy (amoxicillin with clavulanic acid/clindamycin) for five days. To decrease postoperative swelling, a massage of the parotid gland was recommended to the patients. 

The initial follow-up of the patients was done two weeks after the sialendoscopic procedure. The patients were given instructions to immediately report any salivary gland swelling exacerbation. In addition, they were requested to make an appointment at our clinics in the event of re-swelling. The second follow-up for all patients was done by telephone one year after the sialendoscopy. In October 2022, we reached all patients or/and their legal guardians by telephone and collected data regarding salivary gland swelling exacerbations since the performed sialendoscopic procedure.

### 2.4. Statistical Analysis

Data are given as means ± standard deviations (SD), percentages, or ranges when appropriate. Statistical analysis was performed using GraphPad Prism 9 (GraphPad Software Inc., San Diego, CA, USA). The outcome of the sialendoscopy was compared with the number of acute episodes one year before and after the procedure using Mann–Whitney U test. The differences were deemed statistically significant at *p* < 0.05. 

## 3. Results

**Figure 1:**
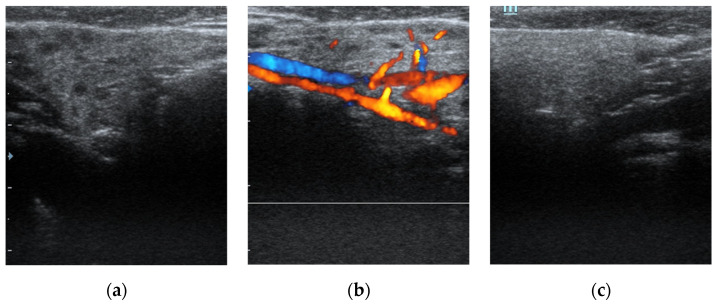
Ultrasound diagnostics of juvenile recurrent parotitis. (**a**) displays a unilaterally affected parotid gland on the right side of a patient. Note heterogeneous parenchyma with several hypoechoic nodules indicating duct dilatation. (**b**) demonstrates increased blood flow of the same parotid gland with Doppler mode. In (**c**), a normal parotid gland on the left side of the same infant is shown as a comparison.

This study included 21 children, of whom eighteen boys and three girls, who were referred to sialendoscopy. There was a preponderance of males with a male-to-female ratio of 6. The mean age of the patient at the time of the procedure was 9.6 ± 5.2 years (range, 2–21). All patients had the US done before the procedure. All clinically symptomatic glands had heterogeneous parenchyma with several hypoechoic nodules observed (Figure 1). 

Thirteen children (62%) had the indications for unilateral sialendoscopy. In contrast, the remaining eight required bilateral procedure—two of the latest group had the second procedure done approximately one year after the first sialendoscopy (Table 1). As a result, the total number of endoscopic procedures was 29. Most endoscopic procedures were performed under general anaesthesia (86%), while local anaesthesia was used on four interventions (14%). Two patients who underwent local anaesthesia had unilaterally involved parotid glands and one had bilaterally affected parotid glands.

**Figure 2:**
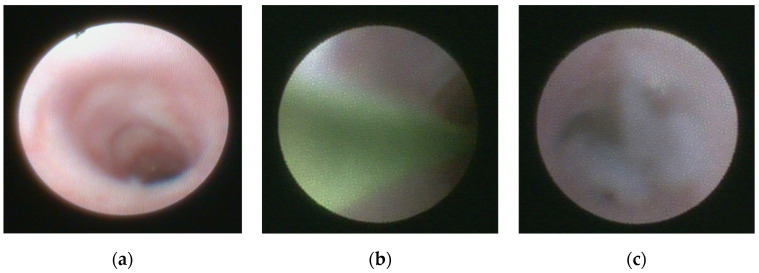
Sialendoscopic appearance of the parotid duct. (**a**) displays the endoscopic appearance of pale mucosa. (**b**) depicts guiding wire introduced during the sialendoscopic procedure to overpass the stenosis. (**c**) shows mucus in the main salivary duct.

During the procedure, the main duct was examined—there was a pale appearance of ductal wall, individual capillaries, and mucous plugs noted to various extent within all patients (Figure 2). Eight patients (38%) had stenosis present and seven had stenosis with a grade of S2 or higher. The mean depth of endoscope introduction into the duct system was 59.1 ± 13.6 mm (range, 22–87 mm). During two procedures, the ductal wall was damaged, therefore we stopped the intervention. In both, steroids were instilled into the duct before this event, and later restitutio ad integrum state was re-established. Consequently, the intraoperative complication rate was noted within 7% of procedures, however, there were no late complications observed or reported.

The patients who underwent local anaesthesia were discharged the same day, while those who had the procedure under general anaesthesia were discharged the following day. Thus, the mean hospital stay was 0.86 days. The antibiotics were administrated to all patients—twenty patients (95%) received amoxicillin with clavulanic acid, and one was given clindamycin due to penicillin allergy. None of the patients had problems with recurrent swelling at the follow-up examination two weeks after the procedure.

Complete absence of swelling of the parotid gland in the year after the procedure was noted in sixteen patients (76%), while five other children (24%) had one episode of gland swelling in the year after the procedure. All relapse episodes had been milder than before the sialendoscopy. A significant decrease in the number of episodes was observed after the procedure (*p* < 0.0001): the mean number of acute episodes in the year before the sialendoscopy was 3.9 ± 2.7, while in the year after the procedure patient had 0.2 ± 0.4 episodes per year. The sialendoscopy was not repeated on the same side of any patient. The mean follow-up time of the patients was 48.6 months (range, 13–116 months). The recurrence of gland re-swelling was reported in only one patient more than twelve months after the sialendoscopy. This patient had four relapses, each episode approximately ten months apart, with the first relapse six months after the sialendoscopy. The episodes of swelling were milder than before the procedure, therefore the re-sialendoscopy was not indicated.

## 4. Discussion

Our study presented a single-centre experience with sialendoscopy in the management of juvenile recurrent parotitis and compared the number of relapse episodes one year before and after the sialendoscopic intervention. Sialendoscopy proved to be an efficient diagnostics and therapeutic method for managing juvenile recurrent parotitis with a significant impact on reducing the number of acute relapses of the disease.

In the past, diverse therapeutic strategies have been employed to manage juvenile recurrent parotitis. The treatment was either conservative or occasionally with aggressive surgical interventions such as Stensen’s duct ligation, superficial or total parotidectomy, and tympanic neurectomy [16]. The current treatment is shifted towards gland preservation with minimally invasive modalities such as sialendoscopy [1,17]. The latest has tremendously lowered the possibility of collateral damage, e.g., facial nerve injury or fibrosis of the salivary gland [4,18]. Intraoperative or postoperative complications of sialendoscopy are rare, namely the postoperative swelling of a parotid gland, iatrogenic perforation leading to swelling of a parapharyngeal space with upper airway obstruction, and perforation of a ductal wall that can result in subsequent duct stenosis [19,20]. The ductal wall perforation was observed in two patients; however, none of them resulted in duct stenosis. The lavage of the duct was immediately stopped, as the instillation might lead to swelling of the parapharyngeal space and could endanger the upper airway.

Given the published reports, the gender distribution of juvenile recurrent parotitis favours males [2,19]. This was also noted in our research. The mean age of patients with juvenile recurrent parotitis at the time of procedure was reported in a more extensive meta-analysis as 7.8 years [19], whereas in our study the mean age of a patient was 23% higher. The latter was caused by three older patients in whom the disease was recognized in late adolescence. We justify this with poor recognition of juvenile recurrent parotitis in primary care and consequently late referral to our centre. As their symptoms began before the age of sixteen, they met the inclusion criteria for this study. It is known that the duct appearance in juvenile recurrent parotitis can share a similar endoscopic appearance in Sjögren’s syndrome; for that reason, this condition must be excluded [21]. In two children with painful swelling in both parotid glands, we observed a changed appearance of the duct wall; however, they both presented with dry eyes and dry mouth. With further diagnostics, Sjögren’s syndrome was proved, therefore, they were not included in this study.

The patients in our study had 3.9 ± 2.7 acute episodes per year before the procedure. This finding is in accordance with several other institutions [22,23,24]; however, few studies present a higher number of swellings, up to 9.2 per year [13]. We hypothesise that this can be attributed to poor knowledge of the disease and subsequently late referral to a specialist in some areas. After the sialendoscopy, the authors report fewer swelling episodes [22,24,25]. Two comprehensive meta-analyses of patients showed a very high percentage of complete resolution of swelling episodes after the sialendoscopy, i.e., 78–81% [19,26]. In accordance with them, our study has a similar result with a 76% of resolution rate in patients. Moreover, after the procedure, the recurrent episodes tend to be less intense, with lower pain intensity. The procedure can be redone if there are several episodes of swellings [27]. In our case series, 5 patients (24%) reported a recurrence of gland swelling one year after the procedure. All of them reported solely one episode per year, therefore, the sialendoscopy was not indicated. Previously, individual institutions performed sialendoscopy on both sides regardless of the involvement of the contralateral gland [28]. More recently, Iordanis et al. noted that children with unilaterally affected parotid glands generally do not require sialendocopic intervention on the contralateral side [25]. Our study supports the previous with only two patients (10%) requiring contralateral sialendoscopy approximately one year after the first.

The majority of research focuses on the follow-up period up to one year after the procedure [3,22,24]. To the best of our knowledge, this study presented the longest follow-up period in patients with juvenile recurrent parotitis after the sialendoscopic procedure [19]. It is noteworthy that solely one patient had relapse episodes reported later than twelve months after the sialendoscopy. As this patient already had one relapse in the first year after the procedure, the overall relapse rate did not differ from the relapse rate within the first year. Considering the previous, most recurrences of the disease were recognized the first year after the procedure then the recurrence rate remarkably decreased.

General anaesthesia is considered optimal for managing salivary gland disease in children [11]. To date, some institutions demonstrated the application of local anaesthesia in children over the age of eight [23,29]. The use of local anaesthesia proved to be a valuable and uneventful option for managing of older children in our centre. The hospital stay of the patients was even shorter, as the patients could leave hospital the same day. The age for local anaesthesia was set at fifteen as this limitation was the patient’s compliance and cooperation; however, we believe that our age limit could be set at a younger age.

The solutions used for the instillation vary between different studies. In our study, dexamethasone was instilled; nonetheless, the instillation of the saline solution alone, saline solution with antibiotics, saline solution with steroids, or saline solution with antibiotics and steroids have already been studied [2,22,24,25,30]. As the solutions were effective regardless of their composition, their main effect is assumingly by breaking the cycle of mucous secretion and stasis by evacuating intraductal mucous plugs and cell debris [16,29]. For that reason, sialendoscopy supposedly addresses the inflammation mainly by cleansing intraductal debris and reopening the stenotic duct [19]. The rate of spontaneous resolution of this disease is unknown [27]. Consequently, the comparison with the group with spontaneous resolutions is not feasible. Although individual researchers consider the resolution by sialendoscopy as over-estimated [27], a considerable reduction of recurrences after the procedure confirms that sialendoscopy is an effective therapeutic tool for disease management. It is important to emphasise that the observed therapeutic effect cannot be attributed exclusively to sialendoscopy because the patients also received intraductal steroid application, antibiotic therapy, and performed salivary gland massage. Thus, the reduction in recurrence rate could be the result of all above mentioned.

We acknowledge that our research has several limitations. The most important limitation of our study is a relatively small sample. Although our study group included most patients with juvenile recurrent parotitis diagnosis in the country, the number of patients was still relatively low due to the rarity of this disease. It is noteworthy that the largest meta-analysis included less than two hundred patients [19], therefore this research considerably extends the sample size. Another limitation is outcomes based on the case series with having no control group. In the future, randomized prospective studies are needed for reaching higher levels of evidence. In addition to previous limitations, all the patients were gathered from a single centre, which might be influenced by local demographics or institutional practice preferences.

## 5. Conclusions

In conclusion, our experience with sialendoscopy had favourable results with the improvement of swelling episodes in all patients. With a mean follow-up time of approximately four years, most patients had relapse episodes observed within the first year after the sialendoscopic procedure. As a relatively safe and minimally invasive technique with a significant impact on the reduction of acute relapse episodes, sialendoscopy emerges as a diagnostic and therapeutic modality of choice in managing juvenile recurrent parotitis in our institution.

## Figures and Tables

**Table 1:** Demographic, clinical, and therapeutic data of children included in this study.

Patient No.	Gender	Age	Side	Stenosis	Anaestesia	Ep. Prior [ep/Year]	Ep. After [ep/Year]	Follow-Up [Months]	Depth [mm]
1	male	21	left	S0	local	7	0	116	70
2	female	16	bilateral: left, right	S0	local	3	0	93	70
				S0	local	2	0	93	75
3	male	3	bilateral: left, right	S1	general	3	1	88	50
				S1	general	3	0	88	50
4	female	20	right	S3	local	2	0	74	55
5	male	5	bilateral: left, right	S4	general	2	0	69	30
				S4	general	4	0	69	22
6	male	5	right	S4	general	5	0	67	40
7	male	10	right	S3	general	4	0	67	50
8	male	12	right	S0	general	3	0	59	70
9	male	6	left	S0	general	10	1	58	50
10	male	6	left	S0	general	5	0	55	56
11	male	14	right	S0	general	3	1	43	63
12	male	12	left	S2	general	4	0	41	65
		13	right	S2	general	2	0	36	63
13	male	13	bilateral: left, right	S0	general	4	0	32	65
				S0	general	2	0	32	65
14	male	4	bilateral: left, right	S0	general	4	0	35	65
				S0	general	3	0	35	65
15	female	10	left	S0	general	2	1	34	75
16	male	2	left	S0	general	4	0	34	65
17	male	6	left	S0	general	4	0	33	73
18	male	13	bilateral: left, right	S0	general	3	0	25	62
				S0	general	2	0	25	60
19	male	6	left	S3	general	3	0	20	50
		7	right	S3	general	2	0	14	52
20	male	9	left	S3	general	15	0	13	52
21	male	7	right	S0	general	3	1	13	87

Stenosis is classified according to the LSD (lithiasis, stenosis, dilatation) classification discussed in this study’s Section 2. Bilateral—both salivary glands had the intervention done in the same setting; depth—depth of the sialendoscope introduction into the duct system in millimetres [mm]; ep. prior/ep. after—number of parotid gland swellings one year before/after the procedure. Follow-up—time in months since the sialendscopic procedure to the check-up in October 2022. Patients 12 and 19 had the contralateral parotid gland affected 9 and 11 months after the first procedure, consequently, the age at the time of the procedure was different.

## Data Availability

The data presented in this study are available on request from the corresponding author.
